# The Effectiveness of Music Therapy on Hand Function in Patients With Stroke: A Systematic Review of Randomized Controlled Trials

**DOI:** 10.3389/fneur.2021.641023

**Published:** 2021-05-25

**Authors:** Wen-Hao Huang, Zu-Lin Dou, Hui-Min Jin, Ying Cui, Xin Li, Qing Zeng

**Affiliations:** ^1^Third Affiliated Hospital of Sun Yat-sen University, Guangzhou, China; ^2^Shanghai Sunshine Rehabilitation Center, Shanghai, China; ^3^Shandong University of Traditional Chinese Medicine, Jinan, China; ^4^Department of Rehabilitation Medicine, Zhujiang Hospital of Southern Medical University, Guangzhou, China

**Keywords:** music supported therapy, hand function, stroke—diagnosis, systematic review, randomized controlled trial

## Abstract

**Objective:** This study aims to evaluate the efficacy of music-supported therapy for stroke patients' hand function.

**Methods:** The databases used included Cumulative Index to Nursing and Allied Health Literature (CINAHL), MEDLINE, PubMed, Embase, Music Index, and Google Scholar. Studies published between January 2010 and August 2020 were included. The searching key terms included “music-supported therapy,” “music therapy,” “hand function,” “hand dysfunction,” “stroke,” “ischemic,” and “hemorrhagic.” Randomized controlled trials or controlled trials involving adults who have hand function problems caused by stroke are included in this study. The methodological quality and risk of bias of the included studies were rated by two independent assessors under the guidance of Cochrane collaboration's risk of bias tool.

**Results:** Twelve studies that met the inclusion criteria were included in this study. Totally, the data included 598 stroke patients (345 male, 253 female) with recruited time from 1.7 months to 3 years, and the mean age of the participants were 61.09 years old. Based on the Cochrane risk of bias tool, study quality ranged from three to seven out of seven points. Compared with the control group, outcomes including hand strength, range of joint motion, dexterity of hands, arm function, and quality of life were significantly superior with music-supported therapy. Five studies reported improved dexterity of hands, and one study reported the improvement of range of motion and strength of patients' hands, which supported the therapy has positive effects on patients' hand function and improving their quality of life after the therapy. The therapy ranged over a period of 4–8 weeks, with an average duration of 30 min/session and an average of three times per week.

**Conclusion:** Based on the results, music-supported therapy could be a useful treatment for improving hand function and activities of daily living in patients with stroke, especially for patients within 6 months after stroke. However, the low certainty of evidence downgrades our confidence to practice in hospital. More and more randomized controlled trials and larger sample sizes are required for a deeper review.

## Introduction

Stroke is believed to affect more than two million patients annually in China and is one of the most common causes of hand function impairment in middle-aged as well as elderly people. The stroke symptoms may include numbness and weakness in the affected arms and cause a loss of coordination and dexterity (1, 2). Although most of the function can be restored with rehabilitation, ranging up to 79%, the recovery of functional problems of the hand left after stroke is not as satisfactory (3). It has been estimated that ~67% of stroke survivors are still unable to use the affected hand 4 years after the onset of stroke (4, 5). Therefore, rediscovering the potential of hand function and improving the quality of life is of great value to stroke patients.

The most commonly used conventional treatment for hand function problems include constraint-induced movement therapy (CIMT), mirror therapy, virtual reality, and music-supported therapy (MST) (6–8). MST for hand function is usually achieved by playing the instructions. The movement patients conduct during playing the piano or grasping drumsticks can facilitate the coordination of hands, strengthen the power of grasp of the impaired hand (9). The aim of the MST is to improve the function of the upper limbs and to provide appropriate stimulation through real-time auditory feedback. Studies have shown that after a 4-week MST program, the hand mobility, fluency, and speed of stroke patients can improve during the test. Besides, the sensory stimulation brought by music can induce functional recovery in damaged hemispheres.

Through the combination of music and movement, MST uses continuous movement and sensory input to enable the patient's central nervous system to re-establish new synaptic connections to the greatest extent possible, thereby creating new neuromotor pathways. Functional magnetic resonance imaging (fMRI) shows that the blood flow of the damaged area of the brain increases when receiving stimulation from MST, which can help repair the cerebral cortex caused by cerebral hemorrhage or cerebral infarction (10). Especially, when patients with high muscle tension caused by stroke, MST can relieve high muscle tension and increase the ability of fingers to move freely. Brain plasticity is associated with treatment-induced recovery, which helps the patient to repair after the brain is damaged (11). When stroke patients participate in MST, they need to process information from multiple senses at the same time, including auditory, visual, and sensorimotor information, which is transmitted from the auditory system to the premotor cortex (PMC), thereby adjusting the top-down output (12). However, a major current focus in MST is to evaluate how does MST works and how does MST helps patients with gait problems (13). Few researchers have addressed the problem of MST improving the hand function of stroke patients.

Up to now, the effect of MST on the recovery of hand function during rehabilitation has not been gone through systematically yet. Therefore, we decided to undertake a systematic review to find out evidence that can support that MST has ideal curative effect in the recovery of impaired hand in stroke patients. The review sheds new light on the therapy for helping patients more effectively and increasing the ability of motor control, especially the hands so that they can finish the daily life task by themselves. One of the main challenges is that we need to search the randomized controlled trials (RCTs) based on MST on stroke patients, which are the gold standard for effectiveness research (14). Our systematic literature review solves the PICOS question, “Does MST can help stroke patients improve their hand function and increase the quality of life?” The answers may provide new thinking for occupational therapy and determine the effectiveness of the MST.

## Methods

This systematic review has been reported according to the Preferred Reporting Items for Systematic Review (PRISMA) statement. A protocol for this review was not registered prospectively.

### Search Strategy

A systematic literature search of the following electronic databases was performed: Cumulative Index to Nursing and Allied Health Literature (CINAHL), MEDLINE, PubMed, Embase, Music Index, and Google Scholar. Databases were searched using a combination of the following keywords considering diagnose, therapy, and outcome. Diagnosis includes stroke, ischemic, and hemorrhagic. Exercise therapy includes music, rhythm, music therapy, MST, music movement therapy, neurologic music therapy (NMT), and neuroscience. The outcome includes neurologic assessment, physical and cognitive disability, strength and dexterity of the hands, gross mobility of hands, and the assessment of quality of life. MEDLINE was searched using MeSH headings which included dystonic disorders, neurofeedback/behavior therapy/exercise therapy, and recovery of function/treatment outcome. Additional articles were identified from reference lists of retrieved articles. The search was conducted in August 2020.

### Eligibility Criteria

Studies were included if they met the following PICOS criteria:

1. Population: The study population included adults (18 years old or older) diagnosed with ischemic or hemorrhagic stroke which was needed to be confirmed by computer tomography or magnetic resonance imaging and by diagnostic guidelines updated by the American Heart Association/American Stroke Association. The patients presenting with the Fugl–Meyer Assessment—upper extremity (FMA-UE) results less than 46 points.
2. Intervention: Interventions had to be sound based, including music listening or listening to rhythmic sequences (MLI or RAS, which could be performed by various instruments, e.g., metronome, synthesizer). At least one group of participants had to perform a task in this condition.
3. Controls: A similar motor act had to be performed without listening to music or rhythmic sequences (control intervention).
4. Outcome: Outcome measures had to assess hand function in a biomechanical manner (e.g., fine motor and gross mobility, strength and dexterity of the hands, functional movements of hands, muscle activity, or muscle-related assessment).
5. Study design: All designs should be randomized controlled trials.
6. Time: Articles published between January 2010 and August 2020.

Studies were excluded from a review when studies were not written in English and if less than half of the participants were musicians. In addition, altered auditory and sensory feedback strategies and studies assessing the combined effects of neuromuscular re-education and transcranial direct current stimulation were also excluded from the review.

### Study Selection

**Figure 1:**
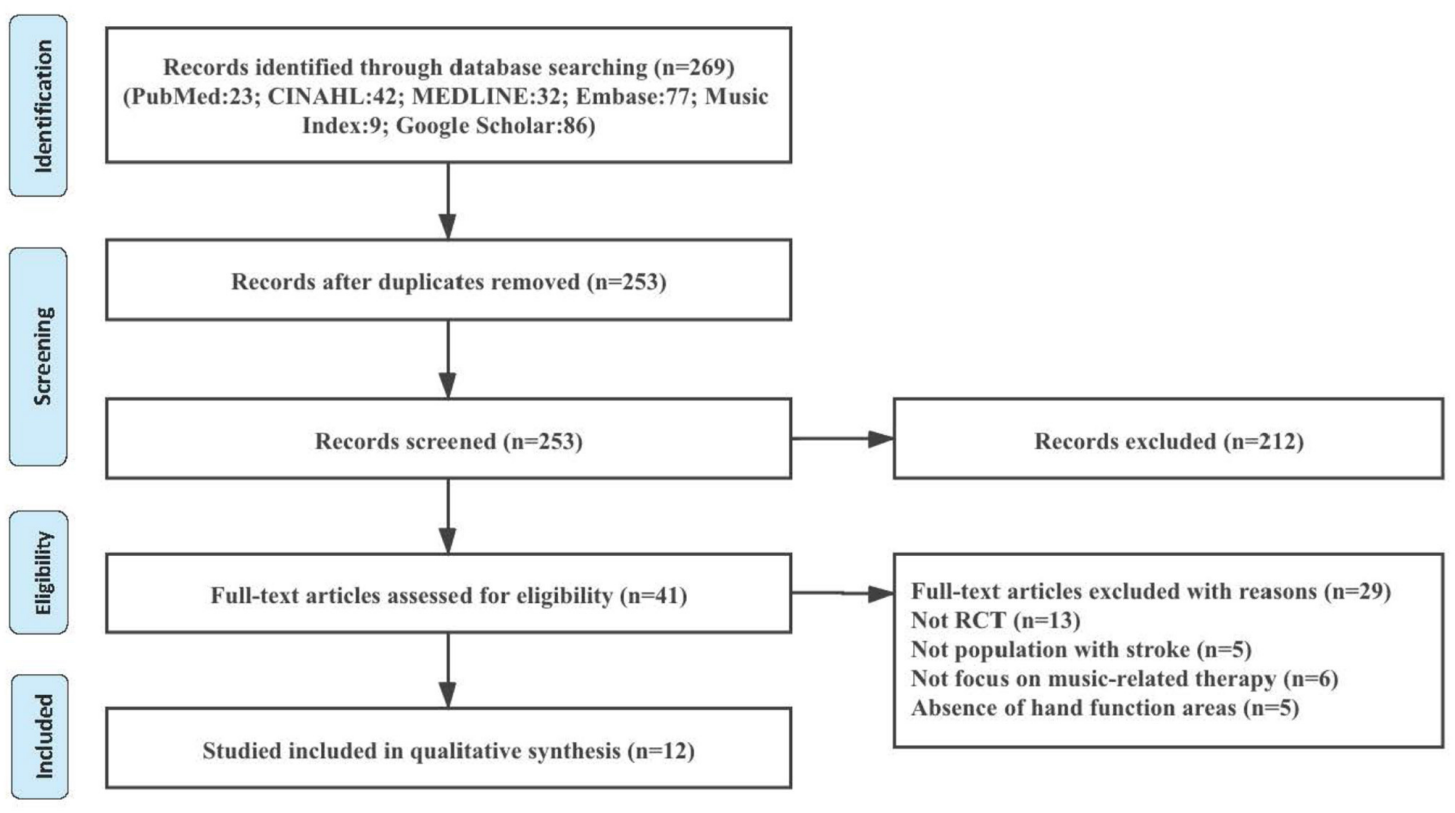
A summary of the PRISMA flow of the study selection process.

The screening procedure was performed by two independent researchers. To collect potentially relevant studies, eligibility was screened based on title and abstract based on the provided inclusion and exclusion criteria described above. Full texts were retrieved and evaluated based on the same eligibility criteria. Afterward, full texts were gathered and evaluated on the previously set inclusion criteria. Reference lists were manually screened to identify additional relevant studies. Results between the two reviewers were compared, in situations where two reviewers were unable to come to an agreement, we took the original articles back together to solve the problem. See Figure 1.

### Data Extraction and Analysis

Data were extracted and documented using an extraction form developed to identify relevant information. Details recorded from each reference included the author's background and discipline, participants, intervention (follow-up), control/comparison, outcome measures, and results. Data and information were extracted by one reviewer and checked for accuracy by a second reviewer. The patients' characteristics and intervention detail of each study were summarized in Table 1, reflecting the heterogeneity of the included studies.

**Table 1:** Summary of the included studies and the detail of intervention and measurement.

**Reference**	**Participants**	**Intervention (follow-up)**	**Control/comparison**	**Outcome measures**	**Results**
Raglio et al. (15)	*N* = 38; age range: 54–89	The standard of care and relational active music therapy approach (*n* = 19) Repeat exercise: 20 sessions lasting 30 min each, 3-weekly	Only standard of care, including physiotherapy and occupational therapy (*n* = 19)	Measured at baseline and at the end of treatment Neurologic: It-NIHSS Physical and cognitive disability: FIM Strength/dexterity of the hands: the Grip-Pinch Dynamometric Test and the Nine-hole Peg Test Gross mobility: TUG Psychological traits and quality of life: HADS and MQOL-It Video-recorded sessions: MTRS	Neurologic: no significant difference between groups Physical and cognitive disability: improved both in experimental and control groups (*p* = 0.001) Strength/dexterity of the hands: the amount of left paretic patients (*n* = 5) improved more than control groups (*n* = 2) Gross mobility: improved both in experimental and control groups (*p* = 0.032) Psychological traits and quality of life: a decrease of anxiety and depression and a significant positive trend
Street et al. (16)	*N* = 11; age range: 53–67	Play acoustic musical instruments and/or iPads with touch screen musical instruments (*n* = 6) Repeat exercise: 20–30 min a session, twice weekly for 6 weeks	Received no intervention (*n* = 5)	Measured at baseline and at 6-, 9-, 15-, and 18-week follow-up Arm function: the action research arm test and the 9-hole peg test	Arm function: no significant difference between groups
Street et al. (9)	*N* = 14; age range: 18–90	Therapeutic instrumental music performance therapy (*n* = 7) Repeat exercise: twice weekly for 6 weeks	Received no intervention (*n* = 7)	Measured at baseline and at 6-, 9-, 15-, and 18-week follow-up Arm function: the action research arm test and the 9-hole peg test Electroencephalography Recording	Arm function: no significant difference between groups Electroencephalography recording: no significant difference between groups
Grau-Sanchez et al. (17)	*N* = 39; age range: 54–92	The regular therapy and extra sessions to play a keyboard and an electronic drum set (*n* = 20) Repeat exercise: 20 individual sessions, 30 min each, 5 sessions/week for 4 weeks	Extra time for exercises for the upper extremity based on the regular therapy (*n* = 20)	Measured at baseline, after the intervention, and at a 3-month follow-up Functional movements: the action research arm test Motor outcomes: FMA and grip strength Fine dexterity: the 9-hole peg test and BBT Activities of daily living: CAHAI Working memory and attention: the digit span subtest from the Wechsler Adult Intelligence Scale III, response inhibition by the Stroop task and processing speed and mental flexibility by the trail-making test Verbal memory: RAVLT and the story recall from the Rivermead behavioral memory test Mood outcomes: the Profile of Mood States, the Beck Depression Inventory Scale, the Positive and Negative Affect Scale, and the Apathy Evaluation Scale. QoL outcomes: the Stroke-Specific QoL Scale and health-related QoL with the health survey questionnaire SF36.	Functional movements: significantly improved functional performance score in the MST group compared with CT group (mean ± SD, standard treatment with exercise, 9.8 ± 7.9, vs. exercise, 6.7 ± 7.9; *p* < 0.001) Motor outcomes: no significant difference between groups Fine dexterity: no significant difference between groups Activities of daily living: no significant difference between groups The cognitive outcomes: no significant difference between groups QoL outcomes: significantly improved in the MST group from baseline to posttreatment compared with CT group (MST group of *t*_(18)_ = −2.23, *p* < 0.05, *d* = 0.54 vs. CT group of no improvements) Mood outcomes: no significant differences between groups in the change scores
Jun et al. (18)	*N* = 30; age range: 54–93	Received music and movement therapy (*n* = 15). Repeat exercise: 1 h/session, 3 times/week for 8 weeks	Received routine care (*n* = 15)	Measured at baseline and at 8-week follow-up Physical functions: range of joint motion Muscle strength: Medical Research Council scale Activities of daily living: K-MBI Mood state: the Korean version of the Profile of Mood States Brief instrument Depression: CES-D	Physical functions: no significant difference between groups Muscle strength: no significant difference between groups Activities of daily living: no significant difference between groups Mood state: the score of experimental group members improved when compared with that of the control group (*t* = 1.818, *p* = 0.040) Depression: no significant difference between groups
Van Vugt et al. (19)	*N* = 34; age range: 30–75	Received its sounds after a random delay sampled from a flat distribution between 100 and 600 ms when the patients play the piano (*n* = 19) Repeat exercise: 10 sessions of half an hour	Received the its sounds immediately when the patients play the piano (*n* = 15)	Measured at baseline, after the intervention Fine motor control: the 9-hole peg test Finger tapping measurements: a triaxial accelerometer (ADXL 335) Mood measurements: POMS	Fine motor control: significantly improved fine motor score in the jitter group compared with normal group (mean ± SD, the average improvement of jitter group, 14 ± 53.6 vs. normal, 3.8 ± 17.9; *p* < 0.001) Tapping speed: no significant difference between groups Tapping variability: no significant difference between groups Mood measurements: no significant difference between groups
Fotakopoulos and Kotlia (20)	*N* = 65; age range: 71–79	A music group (MG) (daily listening to experiential/traditional music) Repeat exercise: 6 months at a frequency of four training sessions/week, of 45 min each session	A control group (CG) with no experiential/traditional music therapy (standard care only)	Measured at baseline, after the intervention Cognitive deficits: mMt Performance in activities of daily living: BI CT perfusion: CBF	Cognitive deficits: significantly improved cognitive score in the recovery group compared with no-recovery group (mean ± SD, the recovery group, 26.38 ± 1 vs. no-recovery group, 24.33 ± 2; *p* < 0.001) Performance in activities of daily living: significantly improved ADL score in the recovery group compared with no-recovery group (mean ± SD, the recovery group, 81.92 ± 2 vs. no-recovery group, 76.53 ± 7; *p* = 0.007) CT perfusion: significantly improved in CBF in affected area in the recovery group compared with no-recovery group (mean ± SD, the recovery group, 29.16 ± 4 vs. no-recovery group, 12.27 ± 11; *p* < 0.001)
Bunketorp-Käll et al. (21)	*N* = 123; age range: 56–70.4	Rhythm-and-music therapy (*n* =41 ) Horse-riding therapy (*n* = 41) Repeat exercise: 2 times a week for 12 weeks	Control group continue with their regular activities and usual care such as outpatient physiotherapy, occupational therapy, or speech therapy (*n* = 41)	Outcome measures were reported at 0 and 6 months postintervention Hand strength: Grippit	Hand strength: significant differences in the mean changes in right-sided maximum and left-sided final grip force Rhythm-and-music group significantly improved their right-sided maximum grip force(16.41 [95% CI, 5.65–27.17]) and left-sided final grip force (17.26 [95% CI, 6.19–28.33]) compared with controls (−1.29 [95% CI, −7.99 to 5.41]) (0.55 [95% CI, −7.07 to 8.17]; *p* = 0.015 and 0.042, respectively); The left-sided improvements were sustained at the 6-month follow-up (*p* = 0.011).
Tong et al. (12)	*N* = 33; age range: 34–64.9	Audible music group (MG) includes conventional rehabilitation treatments and extra sessions of audible musical instrument training (*n* = 15) Repeat exercise: 20 extra sessions over 4 weeks	Mute music group (CG) includes conventional rehabilitation treatments and extra sessions of “mute” musical instrument training (*n* = 18) Repeat exercise: 20 extra sessions over 4 weeks	Measured at baseline, after the intervention Motor function: WMFT, FMA	Motor functions of upper limbs: significant improvements Significant differences in the WMFT were found between the two groups (WMFT-quality: *p* = 0.025; WMFT-time: *p* = 0.037) but not in the FMA (*p* = 0.448). Subjects in MG demonstrated greater improvement than those in CG.
Schneider et al. (22)	*N* = 77; age range: 41.2–68	Music-supported therapy in addition to conventional therapy (*n* = 32). Repeat exercise: 30 min each unit in duration, totally 27.4 units, over 3 weeks	Conventional treatment only (*n* = 30), Without specific additional selection criteria (*n* = 15) standard therapies (physical therapy and individual occupational therapy) 30 min each unit in duration. TG: 28.0 units over 3 weeks CG: 27.2 units over 3 weeks	Measured at baseline, 3-week intervention Motor functions: BBT, the 9-hole peg test, action research arm test, arm paresis score Motor test/parameter: frequency (FREQ), Number of inversions of velocity profiles, Average maximum angular velocity in °/s	BBT, the 9-hole peg test, action research arm test, and arm paresis score: significant improvements in groups TG and MG. Conventional physiotherapy in CG did not produce an improvement, differences between MG, CG, and TG were highly significant, *F*(2, 66) = 6.66, *p* = 0.002. BBT: MG increased the number of cubes grasped by around 10/min. Differences between MG, CG, and TG were highly significant, *F*_(2, 74)_ = 57.08, *p* < 0.001. FREQ: Increase in MG but not TG and CG
Fujioka et al. (23)	*N* = 29; age range: 54.3–64.2	Music-supported therapy used an electronic keyboard and a series of eight electronic drum pads (*n* = 14). Repeat exercise: 30 h of training over 10 weeks	Conventional physical training (*n* = 14) Repeat exercise: 30 h of training over 10 weeks	Measured at baseline, after 5 weeks, after 10 weeks, and 3 months after training completion. Arm and hand subsections of the CMSA Impairment Inventory, action research arm test, BBT	CMSA: Both showed only minor changes over the time course of treatment, hand score was improved at the post 2 time point compared with pre [*t*_(27)_ = −2.27, *p* = 0.031]. A tendency for such improvement was found for the MST group [*t*_(13)_ = −1.88, *p* = 0.082]. The improvement in the GRASP group was not significant. Action research arm test: in the MST group, the decrease between pre and post 2 time points approached significance [*t*_(13)_ = 2.10, *p* = 0.056]. BBT: not to analyze, as eight participants were unable to perform the test at any time point using their affected hand.
Bunketorp-Käll et al. (24)	*N* = 123; age range: 56–70.4	Rhythm-and-music therapy (*n* = 41) Horse-riding therapy (*n* = 41) Repeat exercise: 2 times a week for 12 weeks.	Control group continue with their regular activities and usual care such as outpatient physiotherapy, occupational therapy, or speech therapy (*n* = 41).	Measured at baseline, after the intervention Motor function: Modified Motor Assessment Scale.	Modified Motor Assessment Scale: The MST group did not produce any immediate gains. 6 months 31 post-intervention, the MST group performed better with respect to time; −0.75 s [95% CI, −1.36 to −0.14]; (*p* = 0.035)

*It-NIHSS, the National Institutes of Health Stroke Scale; FIM, the Functional Independence Measure; HADS, the Hospital Anxiety and Depression Scale; MQOL-It, the Italian version of McGill Quality-of-Life Questionnaire; TUG, the Timed Up and Go Test; MTRS, the Music Therapy Rating Scale; mMt, the mini mental test; BI, the Barthel Index; CBF, cerebral blood flow; CMSA, the Chedoke–McMaster Stroke; CAHAI, the Chedoke Arm and Hand Activity Inventory; RAVLT, the Rey auditory verbal learning test; K-MBI, Korean-modified Barthel index; CES-D, The Center for Epidemiologic Studies Depression Scale; POMS, the Profile of Mood States; WMFT, Wolf motor function test; FMA, Fugl–Meyer assessment; BBT, Box and Block test*.

**Figure 2:**
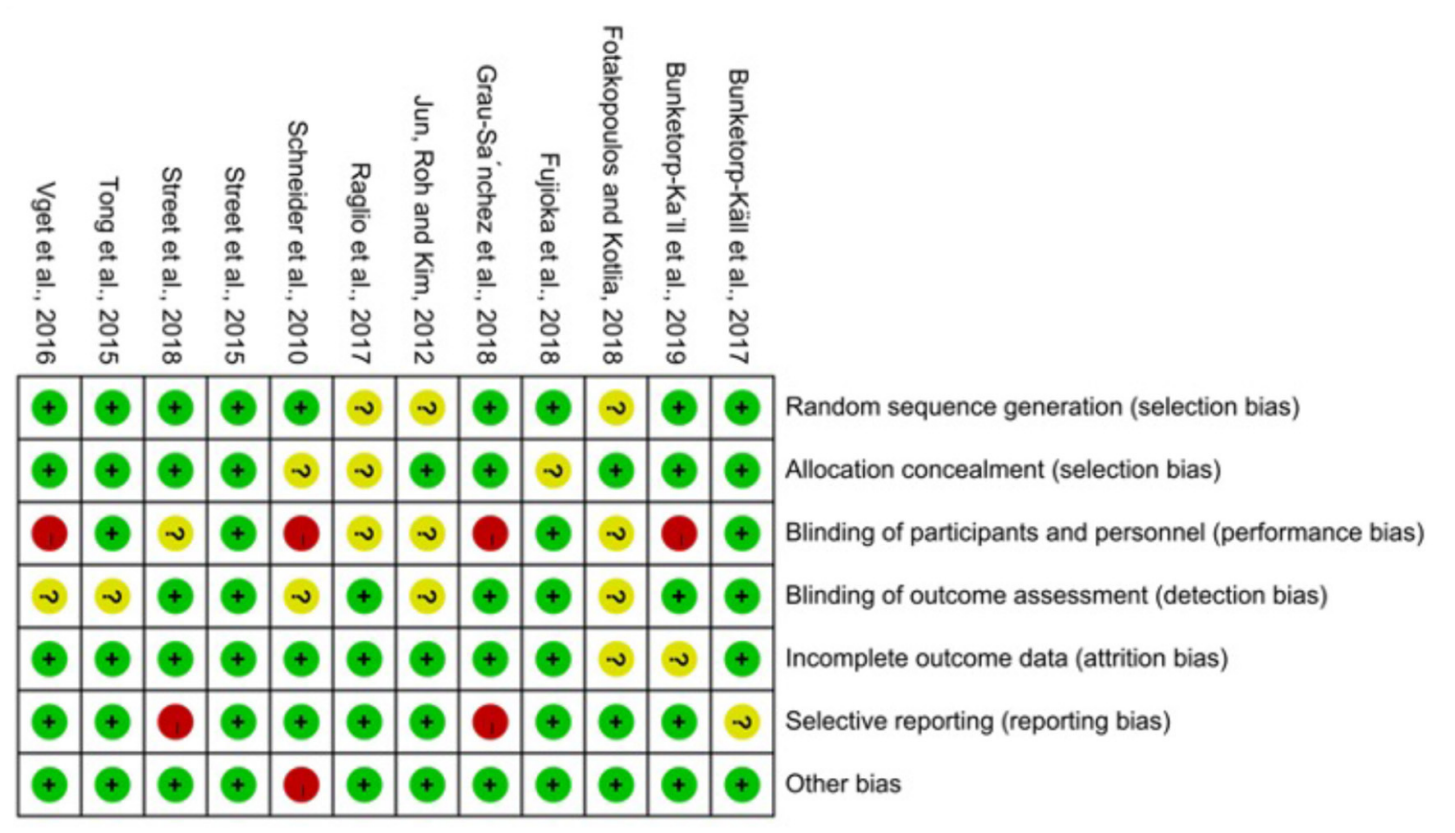
Risk of bias summary.

**Figure 3:**
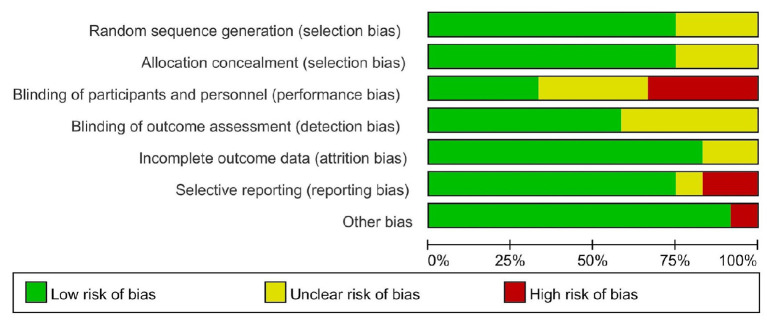
Risk of bias graph.

The risk of bias assessment was based on the handbook of Cochrane (5.1 version). To assess bias, two reviewers independently followed the steps to choose low risk and high risk; if a question was impossible to answer because the original article did not specify it or it was unclear, we chose “unclear.” Reviewers assessed selection (random sequence generation and allocation concealment), performance (blinding of participants and personnel), detection (blinding of outcome assessors), attrition (incomplete outcome data), reporting (selective reporting), and other sources of bias. If there is a disagreement between two reviewers, a third reviewer will solve it. See Figures 2, 3.

## Results

### Study Characteristics

In order to identify the review, a total of 269 articles were retrieved in this search. Two hundred twelve articles were considered for screening, and 41 full-text articles were excluded because the study did not focus on the stroke patients (*n* = 5), the type of study did not meet our inclusion criteria (*n* = 13), the abstract of study did not focus on the music-supported therapy (*n* = 6), and hand function areas (*n* = 5). Twelve articles were included in the systematic review. See Table 1.

### Baseline of Patients

Totally, the data involved 598 stroke patients of mixed gender population. The included studies had the gender distribution as follows: 253 females and 345 males. The mean age of the participants was 61.09 years old (SD = 11.43), ranging from 48.6 to 76.02 years old (9, 12, 16, 17, 19, 21–24). All subjects suffered from stroke from 1.7 months to 3 years before exposed to the MST intervention. Four articles did not provide the time of poststroke of the population in their articles. Based on the data of demographics, the percentage of ischemic stroke was 74.53% (199 participants) and the percentage of hemorrhagic stroke was 25.47% (68 participants).

### Risk of Bias of Included Studies

The items' random sequence generation, allocation concealment, incomplete outcome data, and other sources of bias were assessed as low risk of bias in most of the included studies (9, 12, 16, 17, 19, 21–24). Blinding of participants and personnel scored high risk of bias or unclear risk in half of the included studies, which is inherent to the intervention (15–20, 22, 24). There are some studies that the blinding of outcome assessment was not described clearly so that they were scored unclear in the detection bias (12, 19, 20, 22).

### Intervention Characteristics

Study objectives varied greatly; music was used to influence grip strength (15, 17, 18, 21), range of joint motion (18), dexterity of the hands (9, 15–17, 19, 22), and arm function (9, 12, 16, 17, 22–24), and demonstrated the changes of activities of daily living (17, 18, 20) and quality of life (15, 17, 23). To determine effectiveness, musical interventions were compared with blank control group (9, 16), standard of care, regular activities (15, 18, 20, 21, 23), conventional rehabilitation treatments (12, 17, 22, 24), and mute music training (19, 24). Conventional rehabilitation treatments such as passive mobilization, stretch and progressive resistance exercises, and task-specific training (17, 22), mute music training such as using a sponge or custom-made pad on the musical instrument to inhibit or delay patients from hearing sounds during the training (19, 24).

Nine trials required participants played rhythmical-melodic musical instruments or digital music equipment, such as xylophones, glockenspiels, drums, bongos, ethnic percussion, piano, iPads with touch screen musical instruments (9, 12, 15–19, 22, 23). The methods included free interactions between patients and music therapists and sang a song while playing musical instruments. Three trials employed prepared music and daily listening to experiential/traditional music (20, 21, 24); two of three trials involved structured R-MT combining listening to music while performing coordinated rhythmic sequences and cognitively (21, 24). Participants were trained to begin with motions of the unaffected side and then the affected upper extremity following a modular therapy regime with a stepwise increase of complexity (17, 18).

Sessions were offered one-to-one with individual participants at home or clinical setting (9, 12, 16, 17, 19, 22, 23) or to small groups (15, 18, 20, 21, 24). Within these sessions, there was consistent treatment “dosage,” lasting a single session of 30 or 45 min each. The duration of interventions was variability ranging from 20 sessions over 3 weeks (15) to four sessions weekly over 6 months (20). Delivery of musical interventions was predominantly provided by experienced music therapists or licensed therapists.

### Qualitative Synthesis: Outcome

The included articles show that MST has a better effect on stroke patients in the acute and recovery phases when compared with the group that received conventional rehabilitation. The effect on stroke patients includes many aspects. See Table 2.

**Table 2:** The result of outcome measures in the included studies.

**Outcome measures**	**Measurements**	**Results**
Muscle strength	Grippit	Improvements were shown at the final of intervention and 6-month follow-up (21)
	Grip-pinch test	Strength of nondominant hand significantly increased (15)
	Medical Research Council scale and grip strength	No significant difference between groups (17, 18)
Range of joint motion	Measuring the ROM of shoulder, elbow joint, and hip joint flexion	Significant increase in shoulder flexion and elbow joint flexion (18)
Dexterity of hands	9-Hole peg test	Improved gradually (9, 15, 16, 19, 22)
	9-Hole peg test and box and blocks test	Both groups improved but no significant differences (17)
	Test of finger tapping measurements	No significant difference between groups (19)
Arm function	Wolf motor function test	Significant differences between the 2 groups (12)
	The action research arm test and arm paresis score	Significant differences between the 2 groups (22)
	The action research arm test	No significant difference between groups (9, 16, 17, 23)
	The Modified Motor Assessment Scale (M-MAS)	No significant difference between groups (24)
Activities of daily living	The Barthel Index	Significant differences between the two groups (20)
	Chedoke Arm and Hand Activity Inventory (CAHAI) or Korean-modified Barthel index (K-MBI)	No significant difference between groups (18, 19)
Quality of life	The Stroke-Specific QoL Scale and health-related QoL with the health survey questionnaire SF36	Significant differences between the 2 groups (17)
	Italian version of McGill Quality-of-Life Questionnaire (MQOL-It)	No significant difference between groups (15)

#### Hand Strength

One trial showed significant differences in the mean changes in both sided maximum and one-sided final grip force, as measured with Grippit. Some improvements were also sustained at the 6-month follow-up (21). In the grip-pinch test, one trial showed that the strength of the nondominant hand significantly increased (15). Two trials showed no significant differences were observed for the group comparisons after treatment or at follow-up (17, 18).

#### Range of Joint Motion

Only one trial showed that the ROM (shoulder, elbow joint, and hip joint flexion) on the affected side of subjects in the experimental group was increased following the music therapy, whereas the ROM of these joints in the control group either decreased or remained the same. There was a significant increase in shoulder flexion and elbow joint flexion (18).

#### Dexterity of Hands

Five trials with nine-hole peg test examined fine motor skills of hands improved gradually (9, 15, 16, 19, 22). One trial showed no significant differences were observed for the group comparisons after treatment or at follow-up; however, the within-group analyses revealed that both groups improved after and at follow-up using nie-hole peg test and box and blocks test (17). In the test of finger tapping measurements, there was no significant difference between groups in areas of tapping speed and variability (19).

#### Arm Function

Significant improvements in motor functions of upper limbs after 4 weeks of treatment; however, we only found differences between the two groups in Wolf motor function test (WMFT-quality: *p* = 0.025; WMFT-time: *p* = 0.037), but found no differences in Fugl–Meyer assessment (*p* = 0.448) (12). Grau-Sanchez's (17) study showed that in 39 included patients, no significant difference between MST groups and control groups was shown after a 4-week treatment. Four trials showed no significant difference between groups in arm function through the action research arm test (9, 16, 17, 23). One of the four trials found the within-group analyses revealed that both groups improved after and at follow-up (17). Another trial also indicated minor changes by the CMSA arm and hand impairment scale (23). However, MST patients in one trial showed a substantial improvement over time compared with other groups of patients in the action research arm test and arm paresis score (22). One trial also did not produce any immediate gains with the Modified Motor Assessment Scale (M-MAS) (24).

#### Activities of Daily Living

No significant differences in effect using Chedoke Arm and Hand Activity Inventory (CAHAI) or Korean-modified Barthel index (K-MBI) (18, 19). Only one trial showed significantly improved ADL score in the recovery group compared with the no-recovery group (20).

#### Quality of Life

One trial showed a significant positive trend in quality of life through the Italian version of McGill Quality-of-Life Questionnaire (MQOL-It), but no clinical differences between groups were found (15). There was a significant improvement in the MST group from baseline to posttreatment compared with the conventional treatment groups among other trials (17). Negative effect of affective functions and quality of life was significantly reduced after intervention (23).

## Discussion

The purpose of this systematic review was to evaluate the effectiveness of MST on hand function improvement in stroke patients. The result shows that MST can be useful in improving hand function and the quality of life in stroke patients.

The subjects of studies we focus are symptoms of unilateral hemiparesis in ischemic and hemorrhagic stroke patients. Through a systematic review, we found that MST can improve hand function in patients. Especially for patients within 6 months after stroke, MST can significantly improve their hand function with different aspects such as dexterity of hands.

However, according to the included studies, only a few studies have clear results indicating that the grasping ability and the dexterity of the finger are improved (9, 15, 16, 19, 22). For example, one study stated that the hand function was improved for both the MST group and the control group, but there were no significant differences between the two groups when compared (15). MST did not show superiority improvement when compared with conventional therapy. At this point, we conclude that it may be caused by the following reasons. Firstly, low-intensity music-supported therapy is not known to cause an effect on the improvement of a patient's ability. Therefore, according to the results of a systematic review, training for at least 30 min a day and five times a week is the suitable intensity that is proven as effective. Secondly, some assessment scales, such as FMA, are not sensitive enough to assess the difference in the area of hand function before and after treatment, while assessment scales like WMFT can. The reasons may be because the movement of MST is similar to the movement of evaluation so that it causes some bias that impacts the result of the assessment. Therefore, when we select the assessment scale, we should choose an appropriate scale that can have a good sensitivity to identify the differences. Finally, several studies stated that for their research, the sample size was not large enough to obtain a significantly different result, which suggests that we should do a larger experiment to show the effect of music-supported therapy in the future.

Among the included studies, MST has been described to improve hand function. Compared with traditional treatment, the patient mainly improved the ability of priming, timing, trajectory, and muscle force requirements for the movements of the upper limbs (16), and because of the characteristics of MST, such as, noninvasive, low cost, convenient, and effective intervention, it has been widely used in practice and accepted by Chinese occupational therapists. When playing a musical instrument, the hands follow the beat of the music and make corresponding movements, which is a kind of stimulation to the damaged part of the brain (25). The musical stimulation can promote the extensive activation of the patient's central nervous system' functional network, increase blood flow in the brain area, and accept more stimulation from the movement (26, 27). Among the included articles, there are descriptions of the use of violin, piano, and drumming instruments for treatment. When the patient's hand grasps or strikes the instruments, the patient's grasping ability can be trained purposefully. When the stroke patients train with the rhythm of music, it can help muscle contraction to become more active, so that the sense of participation, rhythm, and speed in the exercise will be more effective (28).

MST can have an improved performance in activities of daily living and enhance the quality of life (17). Furthermore, MST decreases a patient's depression and helps deal with the emotional stress caused by sudden and severe neurological diseases (19). MST helps the patient facilitate their emotions and share their feelings. At the same time, music also plays an important role in motivating patients and stimulating their inner motivation. MST increases patients' motivation due to happiness and intrinsic motivation. We can help them acquire a new skill as well as a hobby so that they can actively participate in MST and enjoy the fun of playing.

## Limitations

The systematic review has some limitations that should be given attention. First, the sample size in the study is not big enough. Studies with larger sample sizes are needed in the coming future. Second, when conducting a systematic search of the literatures, only studies written in English were searched. It is possible that we missed articles of high significance written in other languages. Third, there was a lack of good way of randomization in the included studies, especially with the blinding of patients. It is clear that patients had knowledge of whether they were receiving MST or not, so there was an inability to avoid a placebo effect as a result of receiving MST. Higher-quality articles are needed to provide ideas on how to avoid the placebo effect, and it will be applied for future research development.

## Conclusion

The review included data from 12 randomized controlled trials to explore the effectiveness of MST on the hand function for patients after stroke. Based on current evidence, this study demonstrated that MST can improve hand function and enhance a patient's quality of life. The rhythm and auditory feedback play a vital part in the treatment of MST. However, more well-described randomized controlled trials are required to prove its efficacy.

## Data Availability Statement

The raw data supporting the conclusions of this article will be made available by the authors, without undue reservation.

## Author Contributions

W-HH, Z-LD, H-MJ, YC, XL, and QZ worked together to complete the manuscript. Z-LD and H-MJ assisted W-HH in document retrieval and screening. YC provided statistical assistance and support. XL and QZ provided opinions on grammar and rhetoric. All authors contributed to the article and approved the submitted version.

## Conflict of Interest

The authors declare that the research was conducted in the absence of any commercial or financial relationships that could be construed as a potential conflict of interest.
